# Supplementary figures and images for: Neutrophil–lymphocyte ratio is prognostic in early stage resected small-cell lung cancer
Source: PeerJ. 2019 Jul 29;7:e7232. doi: 10.7717/peerj.7232 (PMC6673426; doi:10.7717/peerj.7232)

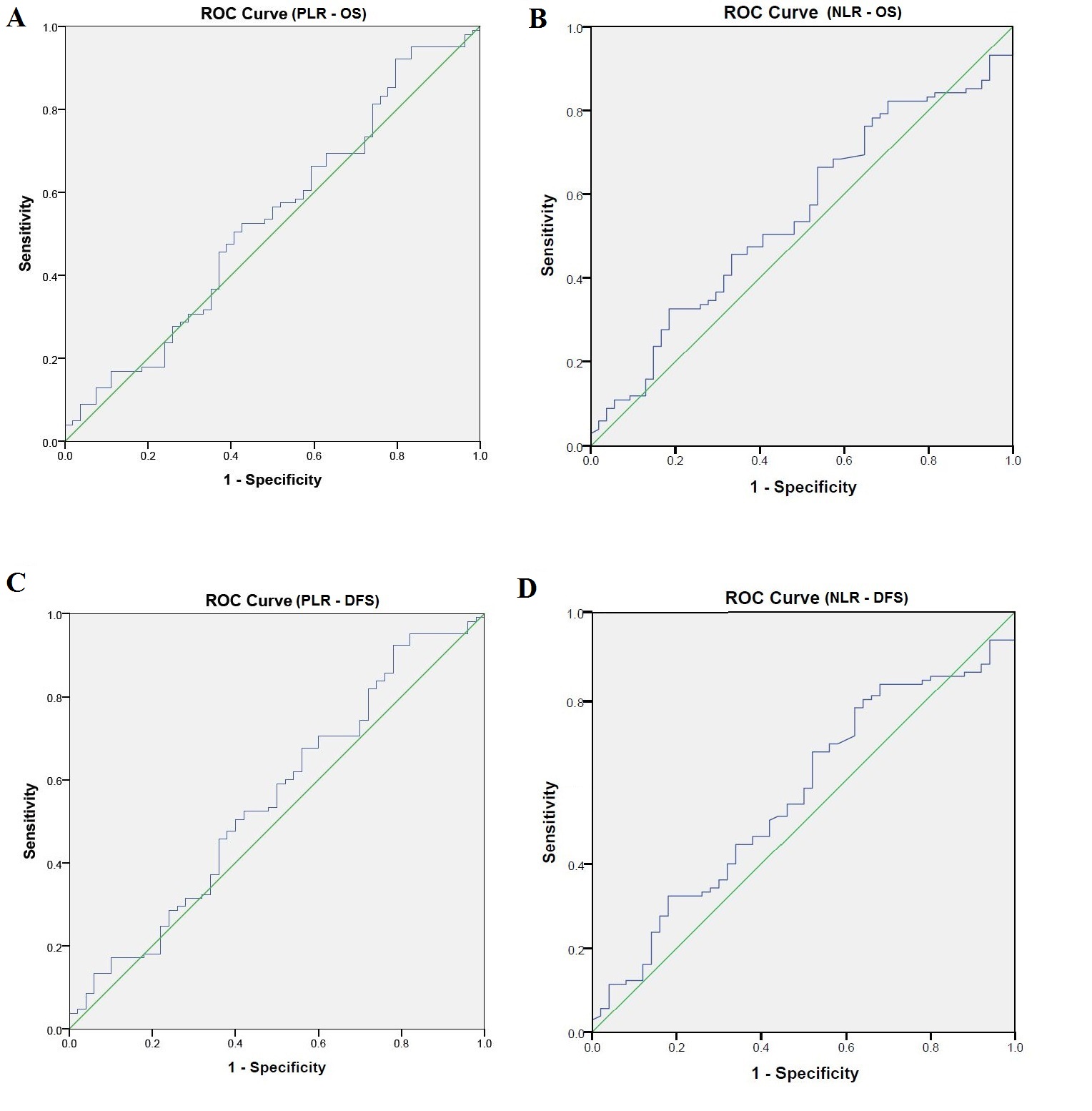

Supplement: Supplemental Information 3 — (A) ROC curve showing optimal cut-off value for PLR in predicting the OS. (B) ROC curve showing optimal cut-off value for NLR in predicting the OS. (C) ROC curve showing optimal cut-off value for PLR in predicting the DFS. (D) ROC curve showing optimal cut-off value for NLR in predicting the DFS. The true positive rate (sensitivity) is plotted in function of the false positive rate (100-specificity). [file peerj-07-7232-s003.jpg]

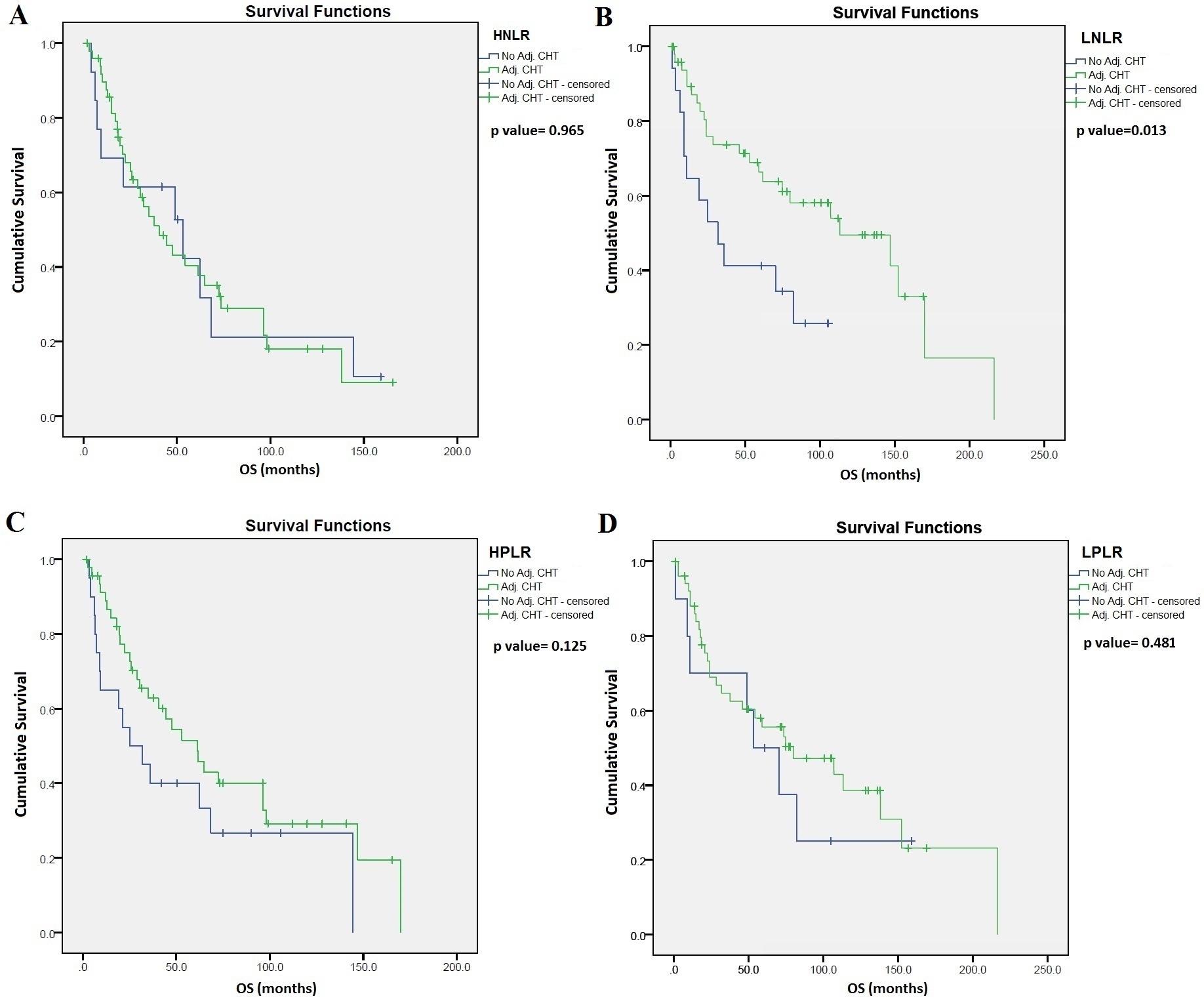

Supplement: Supplemental Information 4 — (A) OS of patients with high NLR (HNLR) (≥2.25) treated with adjuvant CHT compared to those did not receive adjuvant CHT (median OS, 53.2 vs. 40.4 months, respectively, p = 0.965, log-rank test). (B) OS patients with low NLR (LNLR) (<2.25) received adjuvant CHT was significantly longer compared to those did not receive adjuvant CHT (median OS, 113.3 vs. 31.7 months, respectively, p = 0.013, log-rank test). (C) OS of patients with high PLR (HPLR) (≥111) did not receive adjuvant CHT compared to those received adjuvant CHT (median OS, 25 vs. 61.3 months, resepectively, p = 0.125, log-rank test). (D) OS of low PLR (LPLR) (<111) patients did not receive adjuvant CHT compared to those treated with adjuvant CHT (median OS, 53.2 vs. 80 months, respectively, p = 0.481, log-rank test). [file peerj-07-7232-s004.jpg]
